# Endoscopic Removal of Intrauterine Contraceptive Device From the Descending Colon: A Case Report

**DOI:** 10.7759/cureus.58626

**Published:** 2024-04-20

**Authors:** Muhammad Shabbir, Mishal A Aljohani, Abdurahman Alfaiz, Msab Aldakheel, Zeeshan Ali

**Affiliations:** 1 Department of Internal Medicine, Shaqra University, Shaqra, SAU; 2 Department of Medicine, King Abdulaziz Medical City, Riyadh, SAU; 3 Department of Clinical Sciences, College of Medicine, Shaqra University, Shaqra, SAU; 4 Department of Internal Medicine, Prince Mohammed Bin Abdulaziz Hospital, Riyadh, SAU; 5 Department of Otolaryngology-Head and Neck Surgery, Khyber Medical University, Peshawar, PAK

**Keywords:** bowel complications, endoscopic removal, colon perforation, iud migration, intrauterine contraceptive device

## Abstract

Intrauterine contraceptive device (IUCD) is a popular method of contraception used worldwide. Although successful, it can get dislodged from its primary position and perforate the uterine wall. Migration to the colon is an uncommon complication. The patient's symptoms may mimic that of irritable bowel syndrome (IBS), including abdominal pain and changes in bowel movements. The correct diagnosis may be missed for long periods of time, leading to unnecessary suffering and potential complications. It is important for healthcare providers to consider the possibility of intrauterine contraceptive device dislodgement and migration while evaluating patients with a history of IUCD presenting with these symptoms, especially if they have a history of IUCD use. We describe a case where an IUCD was found to be dislodged in the colon and successfully removed through colonoscopy. This case highlights the importance of thorough investigation and follow-up in cases of suspected IUCD migration, as well as the potential for endoscopic removal as a safe and effective method for extracting migrated IUCDs in the bowel.

## Introduction

Intrauterine contraceptive device (IUCD) is one of the most popular and effective methods of contraception, providing long-term reversible birth control [1]. This small device, typically made of copper or hormonal material, is inserted into the uterus and can be left in place for several years, providing effective contraception. However, there have been cases where the IUCD becomes dislodged or migrates to other parts of the abdomen, including the colon [2]. While this is a rare occurrence, it is important to be aware of the potential complications and to take appropriate action if needed. The migration of an IUCD into the colon is an uncommon but serious complication. Reports suggest that the IUCD can potentially migrate into the small or large bowel, including the sigmoid colon. This dislodgement can occur in cases of interval IUCD insertion, and there have even been reports of migration into surrounding organs such as the gallbladder, appendix, and iliac vein. Although rare, abdominal dislodgement of a postplacental IUCD has also been documented, with the device found in and around the uterine wall [3,4].

Symptoms of IUCD migration into the colon can vary depending on the location and extent of the dislodgement. Some patients may remain asymptomatic, while others may experience vague generalized abdominal pain. In rare cases, the IUCD may cause bowel perforation, leading to more severe symptoms such as severe abdominal pain, fever, bleeding, and signs of peritonitis. To diagnose the migration of the IUCD into the colon, initial investigations such as ultrasound and X-ray of the abdomen and pelvis can be performed. These tests can help identify the presence and location of the migrated IUCD. However, for a more accurate localization, a computed tomography (CT) scan of the abdomen and pelvis may be necessary. This can aid in planning the appropriate intervention for extraction. We present a case of an IUCD dislodgement and subsequent migration into the colon, which was successfully removed through colonoscopy.

## Case presentation

**Figure 1:**
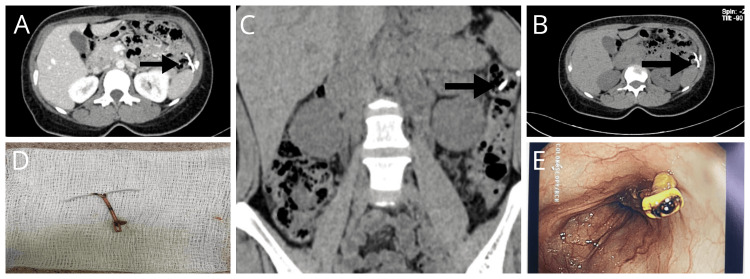
CT of the abdomen showing the IUCD in the descending colon (A, B, and C), the IUCD removed (D), and hemostasis secured endoscopically (E) CT: computed tomography, IUCD: intrauterine contraceptive device

**Figure 2:**
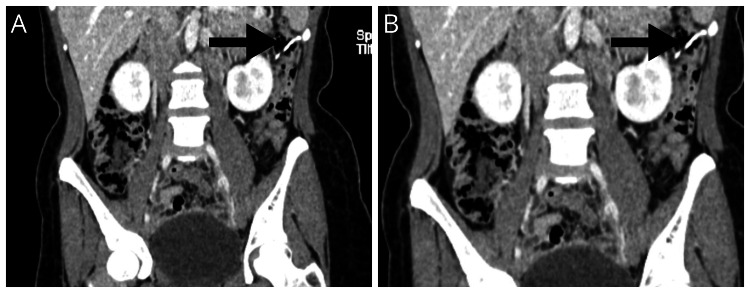
CT of the abdomen coronal section showing the IUCD in the descending colon (arrow) CT: computed tomography, IUCD: intrauterine contraceptive device

A 36-year-old female patient presented to the outpatient department with symptoms of irritable bowel syndrome (IBS), including abdominal pain, bloating, and changes in bowel movements for the last year. The patient was initially evaluated for irritable bowel syndrome but did not show significant improvement with conservative management. Long-standing non-resolving symptoms prompted further investigation, including imaging studies. Ultrasound and X-ray imaging are the primary modalities used to investigate; both of these investigations were performed in another clinic without imaging/films provided to the patient. The reports showed missing IUCD from the uterine cavity. The patient's abdominal symptoms, frequent physician switchovers, and changing consultations were the risks of the patient ending up with some serious complications. Computed tomography (CT) of the abdomen was thus considered appropriate and safe. Abdominal CT was performed to identify any organic abdominal pathology. CT of the abdomen in transverse sections (Figure 1A and Figure 1B) and coronal sections (Figure 1C, Figure 2A, and Figure 2B) revealed the presence of IUCD in the descending part of the colon. Given the potential complications and persistent symptoms, the decision was made to remove the IUCD via colonoscopy. During the colonoscopy, the migrated IUCD was successfully localized and removed (Figure 1D) without any complications. An endoscopic endoclip was applied at the IUCD penetration site after removal to secure hemostasis (Figure 1E). The patient experienced significant improvement in symptoms following the removal of the IUCD.

## Discussion

Dislodgement of IUCD as a complication is a rare occurrence, but when it does happen, it can lead to migration into adjacent structures such as the colon [2]. Symptoms of IUCD migration into the colon can mimic those of other gastrointestinal conditions, such as irritable bowel syndrome, making it challenging to diagnose and treat the underlying cause [5,6]. Furthermore, the presence of adhesions and chronic inflammation surrounding the misplaced IUCD can contribute to persistent symptoms even after removal [7]. In cases where IUCDs migrate into the colon, early diagnosis and prompt intervention are crucial to prevent further complications such as bowel perforation or penetration into adjacent organs [2]. In this particular case, the patient's symptoms of irritable bowel syndrome (IBS) initially led to a misdiagnosis. However, the persistence of symptoms despite conservative management prompted further investigation, ultimately leading to the detection of the migrated IUCD in the descending colon. It is important for healthcare providers to consider the possibility of IUCD migration when evaluating patients with a history of IUCD insertion having chronic urinary or gastrointestinal symptoms [8].

Imaging studies, including ultrasound, X-ray, and CT scans, play a vital role in diagnosing the migration of IUCDs and determining their location. The diagnostic yield of imaging in detecting displaced IUCDs is high, with CT scan being particularly useful in precisely localizing the migrated device [9]. Pelvic and abdominal ultrasound is usually the first line of investigation due to its safety and feasibility. X-ray of the abdomen can locate the site of dislodged IUCD [10]. CT is the most suitable modality for evaluating complications associated with intra-abdominal IUCDs, such as visceral perforation, abscess formation, and intestinal obstruction [11]. The extraction of migrated IUCDs can be challenging, especially when they are embedded in the lumen of hollow organs such as the colon or small bowel loops [12]. In such cases, minimally invasive techniques, endoscopic or laparoscopic removal, are preferred approaches but may require conversion to laparotomy in 34.6% of cases [13].

Endoscopic removal, particularly through colonoscopy, has emerged as a safe and effective technique for removing migrated IUCDs from the colon [14]. It allows for direct visualization and removal of the migrated IUCD without the need for invasive surgery [15]. Studies have shown that endoscopic retrieval is successful in the majority of cases, with a lower risk of complications compared to surgical methods such as laparoscopy or laparotomy [14].

In addition to the advantages of endoscopic removal, it is important to consider the potential risks and complications associated with leaving a migrated IUCD in place. Even in asymptomatic patients, removal of the ectopic IUCD is advisable to prevent future complications such as bowel perforation or fistula formation [6].

## Conclusions

It is important to consider the possibility of IUCD migration in patients with gastrointestinal symptoms who have a history of IUCD use. In these cases, initial investigations such as ultrasound and X-ray can provide helpful information, but a CT scan is often needed for more precise localization. Endoscopic removal through colonoscopy is a safe and effective technique for extracting migrated IUCDs from the colon. With the potential risk for serious complications such as bowel perforation, timely diagnosis is crucial. Confirmation with imaging studies followed by endoscopic removal via colonoscopy is the most appropriate and proactive approach that ensures the safety and well-being of patients. Lastly, it is important to consider endoscopic intervention even in the absence of overt symptoms, because migrated IUCD can cause potential adverse complications.
